# Hypoxia-Inducible Factor-1: A Novel Therapeutic Target for the Management of Cancer, Drug Resistance, and Cancer-Related Pain

**DOI:** 10.3390/cancers14246054

**Published:** 2022-12-08

**Authors:** Bich Phuong Bui, Phuong Linh Nguyen, Kyeong Lee, Jungsook Cho

**Affiliations:** College of Pharmacy and Integrated Research Institute for Drug Development, Dongguk University–Seoul, Dongguk-ro 32, Ilsandong-gu, Goyang 10326, Gyeonggi, Republic of Korea

**Keywords:** hypoxia, hypoxia-inducible factor-1 (HIF-1), tumor microenvironment, drug resistance, cancer-related pain, HIF-1 inhibitors

## Abstract

**Simple Summary:**

Accumulating evidence indicates that hypoxia-inducible factor-1 (HIF-1) plays a pivotal role in tumor biology, particularly in hypoxic environments. Over the past few decades, a number of HIF-1 inhibitors have been identified as potential therapeutic agents for various cancers. However, none of these inhibitors have been successfully translated into clinically available cancer treatments. This review describes the HIF-1 pathway and its roles in tumor proliferation, angiogenesis, and metastasis. In addition, the implications of HIF-1 in the development of drug resistance and cancer-related pain are explored. Finally, the current status of HIF-1 inhibitors in clinical trials and their future perspectives are highlighted, along with their modes of action. This review provides new insights into anticancer drug development targeting HIF-1. HIF-1 inhibitors may be promising combinational therapeutic interventions to improve the efficacy of current cancer treatments and reduce drug resistance and cancer-related pain.

**Abstract:**

Hypoxia-inducible factor-1 (HIF-1) is a key transcription factor that regulates the transcription of many genes that are responsible for the adaptation and survival of tumor cells in hypoxic environments. Over the past few decades, tremendous efforts have been made to comprehensively understand the role of HIF-1 in tumor progression. Based on the pivotal roles of HIF-1 in tumor biology, many HIF-1 inhibitors interrupting expression, stabilization, DNA binding properties, or transcriptional activity have been identified as potential therapeutic agents for various cancers, yet none of these inhibitors have yet been successfully translated into clinically available cancer treatments. In this review, we briefly introduce the regulation of the HIF-1 pathway and summarize its roles in tumor cell proliferation, angiogenesis, and metastasis. In addition, we explore the implications of HIF-1 in the development of drug resistance and cancer-related pain: the most commonly encountered obstacles during conventional anticancer therapies. Finally, the current status of HIF-1 inhibitors in clinical trials and their perspectives are highlighted, along with their modes of action. This review provides new insights into novel anticancer drug development targeting HIF-1. HIF-1 inhibitors may be promising combinational therapeutic interventions to improve the efficacy of current cancer treatments and reduce drug resistance and cancer-related pain.

## 1. Introduction

Cancer is the second major cause of death worldwide, and its incidence is rapidly increasing [1]. For decades, oncologists have continuously endeavored to improve the efficacy and safety of cancer treatments. Various anticancer interventions, including chemotherapy, radiation, and immunotherapy, have been introduced into clinical situations, yet most of the current conventional cancer treatments are found to exhibit certain limitations mainly due to insufficient efficacy, unwanted side effects on the tumor-bearing host, or the development of resistance to the therapy [2,3]. In an attempt to overcome these obstacles, considerable attention has been paid to identifying and characterizing novel and unique molecular targets involved in the regulation of tumor cell proliferation, angiogenesis, and metastasis. Thus, drugs for this targeted therapy deserve particular attraction due to their specificity toward cancer cells while sparing toxicity to non-target host cells [4,5].

Hypoxia (low oxygen level) is one of the hallmarks of the tumor microenvironment. The hypoxic tumor microenvironment of solid tumors is developed due to transient fluctuations in the blood flow (acute hypoxia) or a deficient oxygen supply (chronic hypoxia) [6]. A large number of target genes involved in the processes of tumor progression are triggered to increase oxygen delivery to the hypoxic environment, decrease oxygen consumption, or activate alternative metabolic pathways [6]. One of the important mechanisms is the activation of hypoxia-inducible factor-1 (HIF-1), as a key transcription factor that regulates the transcription of many genes responsible for the adaptation, survival, and aggressiveness of tumor cells [7,8,9]. HIF-1 is also known to facilitate cancer promotion by regulating natural killer cell-mediated antitumor responses and preventing cancer cells from cytotoxic T-lymphocytes induced in the tumor microenvironment [10]. Thus, HIF-1 may affect tumor cell survival by directly and indirectly influencing cancer progression and immunity [4]. Moreover, the overexpression of HIF-1 is associated with poor prognosis and the development of resistance to chemo/radiotherapy in multiple types of human cancers [11,12,13]. From the outcomes of phase II and III clinical trials of HIF inhibitors in the treatment of several types of cancers [14], it is evident that HIF-1 is a promising target for the development of novel cancer therapy. However, none of these inhibitors have thus far been successfully translated into clinical treatments. Therefore, it is necessary to gain an in-depth understanding of the molecular mechanisms by which HIF-1 regulates tumor biology, as well as the benefits and warrants of the current HIF inhibitors.

In this review, we briefly introduce the regulation of the HIF-1 signaling pathway and summarize its roles in tumor progression. In addition, we explore the implications of HIF-1 in the development of drug resistance and cancer-related pain: the most commonly encountered obstacles during conventional anticancer therapies. Furthermore, the current status of HIF-1 inhibitors in clinical trials and their modes of action and perspectives are highlighted in this review. This review may provide new paradigms for treating cancers with HIF-1 inhibitors, either as a single agent or in combination with other conventional therapeutic agents, to improve therapeutic efficacy and minimize drug resistance and cancer-related pain.

## 2. Regulation of HIF-1 Activity in Normoxic and Hypoxic Conditions

HIFs are heterodimeric transcription factors belonging to the basic helix-loop-helix Per/ARNT/Sim (PAS) family that induce the transcription of various genes in response to the cellular oxygen tension [15]. HIFs are composed of a hypoxically inducible α subunit and a constitutively expressed β subunit [15,16,17]. There are three isoforms of the α subunit in humans: HIF-1α, HIF-2α, and HIF-3α. Extensive investigations have focused on the functions of the different α subunits in various diseases, such as cancers that frequently overexpress the α subunits [17]. In this review, we focus on HIF-1α as the most well-studied isoform in tumor biology.

**Figure 1:**
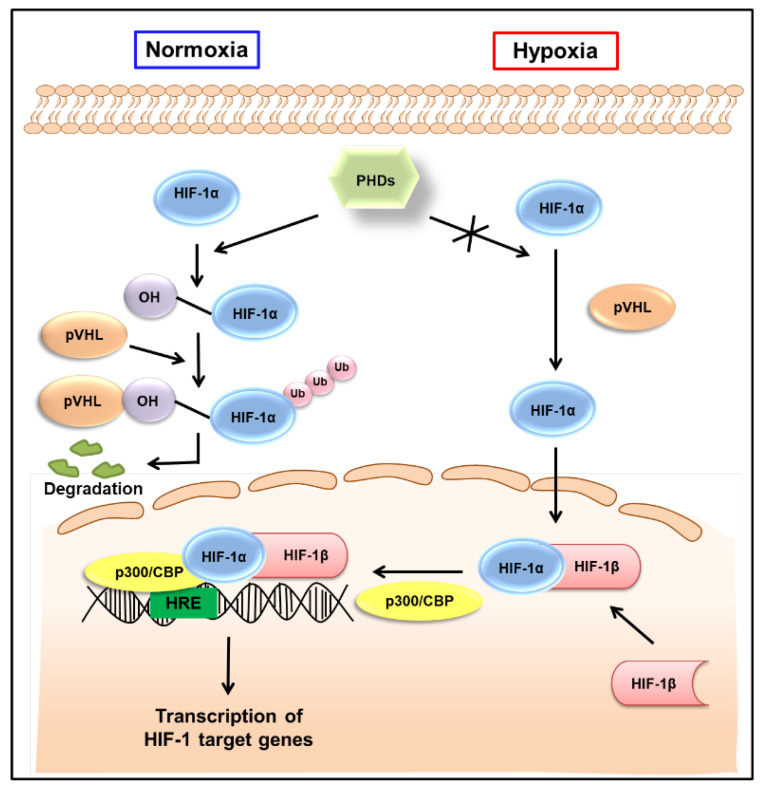
Regulation of HIF-1 activity in normoxic and hypoxic conditions. Under normoxic conditions, HIF-1α is hydroxylated on prolyl residues by PHDs, allowing pVHL to recognize and interact with HIF-1α, eventually leading to the degradation of HIF-1α. By contrast, HIF-1α is stable in hypoxic conditions. It is translocated to the nucleus, where, together with HIF-1β, it binds to hypoxia-responsive elements (HREs) and exerts its transcriptional activity. HIF-1—hypoxia-inducible factor-1; PHDs—prolyl hydroxylase domains; pVHL—von Hippel−Lindau tumor suppressor protein; Ub—ubiquitination; CBP—cyclic adenosine monophosphate response element binding protein.

The HIF-1 signaling pathway has been extensively reviewed elsewhere [18,19]. As shown in Figure 1, HIF-1α is hydroxylated by the prolyl hydroxylase activity of the prolyl hydroxylase domain (PHD)-containing proteins in normoxic conditions, allowing von the Hippel−Lindau tumor suppressor protein (pVHL) to interact with HIF-1α, eventually leading to its degradation. By contrast, HIF-1α is stable in hypoxic conditions. It is translocated to the nucleus and, together with HIF-1β, interacts with the transcriptional coactivator p300/cyclic AMP response element-binding protein (CBP) and binds to the hypoxia-responsive elements (HREs) in DNA to induce the transcription of target genes (Figure 1). The HIF-1 target genes are well-known to be involved in tumor progression, promoting tumor cell growth, vascularization, and metastasis [7]. As HIF-1β is constitutively expressed in the nucleus, the transcriptional activity of HIF-1 primarily depends on the expression and activation of HIF-1α [20]. Therefore, HIF-1α has been considered a promising target to interfere with many aspects of tumor progression.

## 3. Roles of HIF-1 in Tumor Progression

HIF-1 has been demonstrated to be involved in the transcriptional activation of essential genes that regulate the critical processes required for tumor survival and progression. In addition, it is evident that HIF-1 plays a key role in innate and adaptive immune responses and inflammation and, thus, is also considered to be a major transcriptional regulator of immunity and inflammation [21]. The molecular mechanism(s) mediating the initiation and progression of tumors by HIF-1-regulated inflammation includes the increased production of pro-inflammatory mediators, such as cytokines and chemokines, and pro-inflammatory transcription factors, such as nuclear factor κB (NF-κB) [22,23]. Due to the complex role of HIF-1 in various inflammatory cells, an extensive description is necessary to characterize the multifaceted link between inflammation and cancer [24,25]. Since HIF-1 has been strongly indicated in a myriad of cancer cell activities during tumor progression, from the fundamental stages of tumor growth and migration to metastasis [26], we focus on how HIF-1 triggers the transcriptional activation of genes that modulate tumor cell proliferation, angiogenesis, and metastasis in this section.

### 3.1. Role of HIF-1 in Tumor Cell Proliferation and Survival

Increasing evidence suggests that, upon stimulation by growth factors, phosphatidyl inositol-4,5-bisphosphate-3-kinase (PI3K) can upregulate the expression of HIF-1α through the activation of protein kinase B (Akt) and the mammalian target of rapamycin (mTOR), a serine/threonine kinase downstream of Akt, in various human cancers [27]. HIF-1α then induces the expression of various growth factors known to promote cell migration, regeneration, and proliferation, such as transforming growth factor-β (TGF-β), insulin-like growth factor 2 (IGF2), endothelin-1 (END-1), erythropoietin (EPO), and macrophage migration inhibitory factor (MIF) [18,28,29]. In addition, growth factor receptors, such as the epidermal growth factor receptor (EGFR), are also known to be induced by HIF-1α [18,28].

Tumor cells exhibit increased metabolic flexibility and adaptability to sustain their cell growth and survival in the tumor microenvironment characterized by relative nutrient deprivation, hypoxia, and hypovascularity. Unlike normal cells, tumor cells tend to turn their metabolism from an oxygen-dependent tricarboxylic acid (TCA) cycle to glycolysis: an oxygen-independent pathway [30]. Glycolysis is a principal route of ATP synthesis in tumor cells and has been comprehensively reviewed [30]. Increased glycolysis is thought to be a natural response to the hypoxic condition of rapidly migrating cells via the HIF-1 pathway [31]. HIF-1 activation increases many glycolytic enzymes, including hexokinase 2 (HK-2) and phosphofructokinase 1 (PFK1), which appear to be oncogenes required to mediate tumor initiation and growth [32,33]. Another glycolytic enzyme, pyruvate dehydrogenase kinase (PDK), is also augmented by HIF-1. The upregulation of PDK1 decreases acetyl-CoA flux into the TCA cycle, subsequently converting the metabolic program from mitochondrial respiration to glycolysis [34,35]. Moreover, the HIF-1-enhanced induction of lactate dehydrogenase (LDH) mediates the conversion of pyruvate to lactate, accompanied by the recycling of cytosolic NAD+, which is necessary for further glycolysis [36]. In addition to glycolysis, HIF-1 also supports the process of glucose uptake and oxidation by inducing the expression of glucose transporters (GLUT), including GLUT1 and GLUT3 [37], which then increase cellular glucose uptake and oxidation. The intermediary metabolites of the glycolytic pathway supply precursors for the synthesis of glycine, serine, purines, pyrimidines, and phospholipids, necessitating HIF-1-regulated metabolic reprogramming for tumor cell growth and maintenance under stress [28]. These modifications by activated HIF-1 ultimately enhance the ability of tumor cells to upregulate glycolysis, protect cells from oxidative damage, and promote ATP production to sustain tumor cell proliferation, even in the absence of oxygen. These findings illustrate the notable efforts to restrain these processes by inhibiting HIF-1. For example, a novel HIF-1α inhibitor, IDF-11774, blocking HIF-1α accumulation under hypoxia in human colon cancer cells has been found to suppress tumor growth by inhibiting the expression of GLUT1 and PDK1 [38]. Recently, the disubstituted adamantyl derivative LW1564 has been reported to impair tumor metabolism by suppressing HIF-1α accumulation, thereby inhibiting the tumor growth of hepatocellular carcinoma cells in vitro and in vivo [39].

Cell proliferation requires the precise spatiotemporal regulation of the intracellular pH [40]. In hypoxia, pyruvate is converted to lactate in a reaction catalyzed by LDH, and the accumulation of lactic acid may result in an acidic environment. Tumors adapt to pH changes and control their normal proliferation function by carbonic anhydrases (CAs), which reversely convert carbon dioxide and water to carbonic acid. The activity of CAIX and CAXII are decreased by *VHL* [41] and are significantly induced in hypoxic conditions [42,43,44], suggesting that their transcription may be regulated by HIF-1. Numerous CA inhibitors are also emerging as promising agents for the treatment of hypoxic tumors by disrupting HIF-1-regulated pH homeostasis [43,44,45]. Thus, regulation of the intracellular pH by HIF-1 also plays an important role in driving tumor proliferation.

A balance between cell proliferation and apoptotic cell death is critical in maintaining biological processes and homeostasis. The dysregulation of this balance has been implicated in multiple types of disease, including cancer [46]. It has been demonstrated that the overexpression of HIF-1α inhibits hypoxia-induced apoptosis in human oral squamous cell carcinoma cell lines via inhibiting cytochrome *c* release from the mitochondria and reactive oxygen species (ROS) generation. An increased expression of anti-apoptotic Bcl-2 and Bcl-XL, as well as decreased levels of pro-apoptotic Bax and Bak, were also observed [47]. Moreover, cobalt chloride, a chemical inducer of HIF-1, reduced the *tert*-butyl hydroperoxide-induced apoptotic death of HepG2 human hepatoma cells, as measured by DNA fragmentation [48]. These data suggest that HIF-1 may exert an anti-apoptotic role in various types of cancer cells.

Autophagy is a highly conserved mechanism in which cell contents are transported to lysosomes for breakdown or are utilized to produce macromolecules for energy synthesis [49]. The evidence suggests that HIF-1α-mediated autophagy in tumor cells supports cell survival but not cell death [50]. It was previously reported that HIF-1 was required for hypoxia-induced mitochondrial autophagy (mitophagy), preventing oxidative phosphorylation during the adaptation of tumor cells to hypoxia [51]. The HIF-1-induced autophagy process was then demonstrated as a survival mechanism by inducing the expression of Bcl-2/adenovirus E1B 19 kDa interacting protein 3 (BNIP3) and a similar protein BNIP3L to disrupt the Bcl-2/Beclin-1 or Bcl-XL/Beclin-1 complex [50]. Another possible mechanism of HIF-1-dependent autophagy involves the p27/E2 transcription factor 1 (E2F1) signaling pathway. HIF-1 positively modulates p27, subsequently repressing E2F1 activity to induce autophagy [52]. The HIF-1α-dependent upregulation of autophagy and cell survival has been postulated in prostate cancer [53], pancreatic cancer [54], and colorectal cancer (CRC) [55].

Collectively, HIF-1 plays crucial roles in tumor cell proliferation and survival. HIF-1 stimulates the expression of many growth factors, such as TGF-β, IGF2, END-1, and EPO, as well as growth factor receptors, such as EGFR. In addition, HIF-1 regulates the metabolic pathways of tumor cells to gain energy and nutrients for survival and growth by promoting aerobic glycolysis and autophagy. The regulation of the intracellular pH and apoptosis by HIF-1 may also contribute to tumor cell proliferation and growth. Further investigation of a wide variety of tumor cell types under different hypoxic conditions may consolidate the roles of HIF-1 in tumor cell proliferation and survival.

### 3.2. Role of HIF-1 in Angiogenesis

Angiogenesis, the physiological process through which new blood vessels form from pre-existing vessels, is also a widely known hallmark of cancers [56]. The sustained expansion of a tumor mass requires new blood vessel formation to rapidly provide proliferative tumor cells with a sufficient supply of oxygen and nutrients [57]. Multiple HIF-1-targeted genes have been shown to modulate angiogenesis [58,59]. Thus, HIF-1 has become an attractive target for cancer therapy [60,61].

HIF-1 activates angiogenesis by stimulating the production of the vascular endothelial growth factor (VEGF) and many other angiogenic factors, such as the placenta-like growth factor (PLGF), platelet-derived growth factor-β (PDGF-β), and angiopoietin (ANG) [62]. For example, HIF-1 binds to HRE within the VEGF promoter, resulting in increased gene transcription. In addition, VEGF mRNA levels are shown to be enhanced by HIF-1 through the regulation of mRNA stability [63]. VEGF then binds to five different receptors: VEGFR-1, VEGFR-2, VEGFR-3, neuropilin-1, and neuropilin-2. VEGFR signaling leads to a cascade of events, including the migration and proliferation of endothelial cells (ECs) and the induction of vascular permeability in tumor vessels [64]. HIF-1α also induces VEGFR-1, -2, and -3 during hypoxia in vascular and lymphatic ECs [65,66].

ANGs are the ligands of the Tie-2 receptor tyrosine kinase. While ANG-1 plays an essential role in embryonic angiogenesis, both ANG-1 and ANG-2 have been demonstrated to be involved in tumor angiogenesis [67,68]. By contrast, ANG-3 was reported to inhibit the tumor angiogenesis and metastasis of Lewis lung carcinoma and TA3 mammary carcinoma cells, probably through the inhibition of ANG-1- and VEGF-induced activation of extracellular signal-regulated protein kinase 1/2 (ERK1/2) and Akt [68]. Using primary human ECs, ANG-4 was demonstrated to be induced by HIF-1 and participate in hypoxia-induced angiogenesis. These findings support the key roles of HIF-1 in the angiogenic processes of different types of tumor cells [69].

Additionally, HIF-1α promotes angiogenesis by regulating the matrix metalloproteinases (MMPs), plasminogen activator inhibitor (PAI), and vascular tone governing nitric oxide synthase (NOS) [70,71]. The MMP-mediated digestion of the extracellular matrix (ECM) initially allowed the proliferative ECs to migrate through the matrix as an essential step for the development of new blood vessels. MMP-2, which correlates with the tumor grade and vascularity in many cancer types, such as human astrocytoma, is shown to be upregulated by HIF-1-dependent pathways in hypoxia [72]. HIF-1 also increased the expression of PAI-1, which plays an important role in the degradation of ECM proteins and the facilitation of cell migration, contributing to angiogenesis [73]. Moreover, in hypoxia, the HIF-1α-induced expression of inducible NOS (iNOS) further increases NO concentration, which contributes to tumor progression by promoting the neovascularization of tumor masses [74]. The relationship between HIF-1α and NO in cancer angiogenesis appears quite complex and poorly understood. Thus, further study is needed for clarification. Taken together, accumulating evidence supports the fact that hypoxia and HIF-1 are the key regulators of blood vessel growth through the upregulation of many pivotal angiogenic factors, particularly VEGF, ANG, MMPs, PAI-1, and NOS.

### 3.3. Role of HIF-1 in Tumor Cell Metastasis

Metastasis is a highly complex process that accounts for the majority of deaths in cancer patients [75]. Hypoxia-induced HIF-1 affects multiple steps within the metastatic cascade, including epithelial−mesenchymal transition (EMT), intravasation, extravasation, and pre-metastatic niche formation [76].

EMT is considered an early step in the metastasis process, which is defined by the loss of epithelial cell−cell adhesion and the acquisition of mesenchymal characteristics. While E-cadherin deficiency lowers cell adhesion junctions and cell polarity, the mesenchymal proteins reorganize the cytoskeleton to facilitate a motile phenotype of the tumor cells [75]. HIF-1 directly induces the transcription of Snail [77], ZEB1 [78], TWIST [79], and TCF3 [80], whose gene products, in turn, repress E-cadherin expression. HIF-1 also indirectly promotes EMT via other signaling pathways, including chemokines [23], NF-κB [81], TGF-β [82], and Notch [83,84] signaling pathways.

Intravasation is the entry of tumor cells into the circulatory system and is required for cell survival in this transit as circulating tumor cells (CTCs). Before penetrating the surrounding interstitial ECM, EMT tumor cells first have to destroy the integrity of the basement membrane (BM). Tumor cells regulate their surface receptors, such as integrins, to form a connection with BM components and invade through this link or secrete collagen-degrading enzymes, such as MMPs [75]. HIF-1 is shown to activate the expression of the urokinase plasminogen activator surface receptor [85] and hepatocyte growth factor receptor [86] to alter the interactions between integrins and ECM. The HIF-1-dependent pathway is also involved in the upregulation of MMP-2 and MMP-9, which are mediators of BM degradation [87]. Furthermore, HIF-1-induced VEGF can facilitate vascular permeability, thereby increasing the chances of intravasation by tumor cells. To survive in the bloodstream, the CTCs must avoid anoikis: a type of apoptosis caused by a lack of integrin attachment to ECM. HIF-1 has been demonstrated to mediate anoikis resistance by suppressing the α5 integrin [88].

Extravasation occurs in a distant organ where the blood vessels are usually formed and nurtured, posing a difficult barrier for tumor cells to overcome [75]. CTCs should adhere to ECs and disrupt their connection with ECs to extravasate at the metastatic site. Hypoxia-induced HIF-1 is considered a stimulant of the extravasation of breast cancer to lung cancer via the HIF-1-mediated L1 cell adhesion molecule (L1CAM) and angiopoietin-like 4 (ANGPTL4) [89]. While L1CAM increases the adherence of breast cancer cells to EC monolayers via hemophilic or heterophilic interactions, ANGPTL4 blocks EC−EC interactions, facilitating the vascular metastasis of breast cancer cells to the lung parenchyma. In addition, the tumor cell extravasation into host organs is also a result of chemokine interaction within the microenvironment of specific organs. The interaction of EC-secreted stromal cell-derived factor-1, also known as C-X-C motif chemokine 12 (CXCL12), and C-X-C chemokine receptor 4 (CXCR4) by HIF-1 under hypoxic conditions serve as a good illustration of this step [90]. The CXCL12/CXCR4 axis is also reported to participate in the extravasation of metastasizing cancer cells [91,92]. Based on these findings, the HIF-1-stimulated CXCL12/CXCR4 axis seems necessary for the extravasation of organ-specific sites.

Finally, the metastatic site must be primed before cancer cell arrival to present a suitable microenvironment for cell survival and colonization at the new distant organs (the pre-metastatic niche) [75]. During pre-metastatic niche formation, bone marrow-derived cells are recruited to metastatic sites where they form cell clusters before tumor cell arrival, and then they are eventually colonized by metastatic cancer cells [93]. The lysyl oxidase (LOX) family remodels collagen cross-linking at the site of the pre-metastatic niche and is essential for the recruitment of bone marrow-derived cells [93,94]. It is speculated that HIF-1 is a master regulator of breast cancer metastatic niche formation to the lungs through the secretion of multiple members of LOX. As a result, ECM increases the tensile strength for focal adhesion formation, thereby enhancing lung cancer cell colonization [93,95].

**Figure 2:**
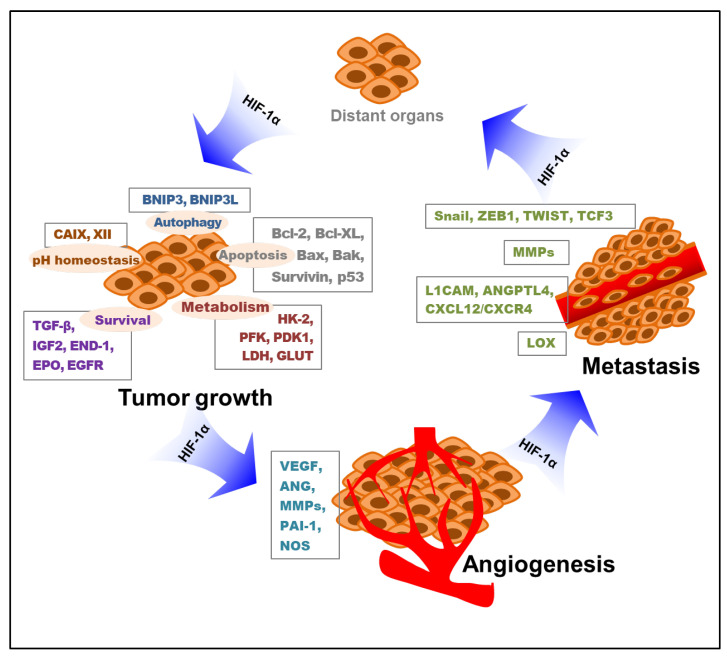
Roles of HIF-1 in tumor growth, angiogenesis, and metastasis. Various targeted molecules and pathways involved in the HIF-1-mediated tumor progress are indicated. HIF-1—hypoxia-inducible factor-1; BNIP3—Bcl-2/adenovirus E1B 19 kDa interacting protein 3; BNIP3L—Bcl-2/adenovirus E1B 19 kDa interacting protein 3-like; CAIX—carbonic anhydrase IX; CAXII—carbonic anhydrase XII; TGF-β—transforming growth factor-β; IGF2—insulin-like growth factor 2; END-1—endothelin-1; EPO—erythropoietin; EGFR—epidermal growth factor receptor; HK-2—hexokinase 2; PFK—phosphofructokinase; PDK1—pyruvate dehydrogenase kinase 1; LDH—lactate dehydrogenase; GLUT—glucose transporter; VEGF—vascular endothelial growth factor; ANG—angiopoietin; MMP—matrix metalloproteinase; PAI-1—plasminogen activator inhibitor-1; NOS—nitric oxide synthase; L1CAM—L1 cell adhesion molecule; ANGPTL4—angiopoietin-like 4; CXCL12—C-X-C motif chemokine 12; CXCR4—C-X-C chemokine receptor 4; LOX—lysyl oxidase; HIF-1α—hypoxia-inducible factor-1α.

Briefly, hypoxia-induced HIF-1 influences all the important processes of metastasis, from the induction of EMT to their survival in circulation and metastatic colonization. This implies a therapeutic angle whereby patients with metastatic cancer with high levels of HIF-1 may benefit from HIF-1 inhibitors. Collectively, hypoxia-stimulated HIF-1 mediates tumor cell proliferation, survival, angiogenesis, and metastasis. The key roles of HIF-1 in these processes of tumor growth, angiogenesis, and metastasis through the modification of various targeted molecules and pathways are depicted below (Figure 2).

## 4. Roles of HIF-1 in Anticancer Drug Resistance and Cancer-Related Pain

### 4.1. Role of HIF-1 in Anticancer Drug Resistance

The overexpression of the HIF-1α protein is associated with the development of resistance to chemo- and radiotherapy in multiple types of human cancers [11,12,96,97,98]. Several molecular mechanisms by which hypoxia and HIF-1 drive tumors to therapeutic resistance are discussed below.

One of the molecular mechanisms clarifying the contribution of HIF-1 in drug resistance is drug efflux which is a major obstacle to effective drug delivery and retention [99]. At least three drug transporters are found to be regulated by HIF-1, including the multidrug resistance 1 protein (MDR1), multidrug resistance-associated protein 1 (MRP1), and breast cancer resistance protein (BCRP). The high expression of the human *MDR1* gene and its encoded transporter, P-glycoprotein (P-gp), can reduce the intracellular concentrations of numerous chemotherapeutic agents, such as vinca alkaloids, anthracyclines, paclitaxel, and doxorubicin, by acting as a drug efflux pump [100,101]. Thus, tumor cells that overexpress *MDR1*/P-gp usually show resistance to chemotherapy. It has been shown that hypoxia-regulated HIF-1α is correlated with the *MDR1*/P-gp expression in many cancer cell lines, such as breast, colon, and bladder cancer cells [99,102,103]. MRP1 is also identified as the cause of multidrug resistance by transporting unmodified hydrophobic compounds, such as anthracyclines [104]. HIF-1 elevated the transcription of the gene encoding MRP1 (*ABCC1*) in response to hypoxia, and the inhibition of HIF-1 by siRNA against HIF-1 significantly reversed this effect in colon cancer [105]. Similar to MDR1 or MRP1, BCRP is also known as an ATP-dependent efflux multidrug transporter, pumping chemotherapeutic agents out of the cells [106]. BCRP is associated with the resistance of breast and colon cancers to chemotherapy and is induced by the hypoxic condition in a HIF-1-dependent manner [107,108].

Another mechanism underlying the development of resistance to anticancer therapy may originate from the capacity of hypoxia-induced HIF-1 to repair DNA damage. DNA damage is the central point of cancer treatment, as the majority of anticancer drugs use this basic mode of action to kill tumor cells. However, tumor cells may respond and attempt to overcome this DNA damage initiated by chemo/radiotherapy through various repair mechanisms [109,110]. Chemo/radiotherapy may induce single-stranded DNA breaks (SSBs) or double-stranded DNA breaks (DSBs) to start apoptosis. The repair of SSBs is carried out by poly(ADP-ribose) polymerase 1 (PARP-1) or by the regulation of the *XPA* and *XPD* genes: the key enzymes involved in the base excision repair process. To repair DSBs, tumor cells may develop the DNA damage response, which is a complex network that targets various DNA lesions [111]. Increasing evidence shows that most molecules in the DNA repair pathway are regulated by HIF-1α. For example, HIF-1α mediates the overexpression of PARP-1, XPA, and XPD, which subsequently leads to the resistance of cisplatin in non-small cell lung cancer (NSCLC) and testicular germ cell tumors [112,113]. HIF-1α also regulates the activities of several kinases involved in the DSBs repair pathway in radiation-treated mouse mesenchymal stromal cells [114]. Additionally, HIF-1α has been shown to inhibit the formation of both radiotherapy-induced DSBs and SSBs, followed by increased radioresistance in hepatocellular carcinoma [115]. These collective results support the emerging role of HIF-1α in promoting DNA repair and chemo/radioresistance in a range of tumor cells.

Moreover, HIF-1α affects chemo/radiosensitivity via the regulation of genes that are related to tumor metabolism. Numerous genetic alterations appear to be involved in metabolic reprogramming in a HIF-dependent manner. The HIF-1α-mediated PDK3 upregulation significantly inhibited cell apoptosis and increased resistance to either cisplatin or paclitaxel in colon cancer [116]. The inhibition of HIF-1α-induced LDH expression can restore the sensitivity to bortezomib in multiple myeloma (MM) cells [117]. Pyruvate kinase M2 (PKM2) is also a transcriptional target of HIF-1α. Thus, it has been proposed that PKM2 inhibition may be used to sensitize hypoxic tumors to chemo/radiotherapy [118]. The stabilization of HIF-1 also increases the expression of carbonyl reductase 1 (CBR1) in hypoxic hepatocellular carcinoma cells and MCF-7 breast cancer cells. CBR1 is an NADPH-dependent enzyme that catalyzes the biogenic transformation of doxorubicin to its metabolite doxorubicinol, which is much less effective against cancer than doxorubicin. In this way, HIF-1 may induce the metabolic reprogramming of cancer cells and desensitize them to anticancer drugs [119].

Resistance to apoptosis, a common feature of tumor cells, is indicated as a promoter of cancer malignancy [120,121]. There is substantial evidence to suggest that HIF-1α can contribute to abnormalities in the apoptosis machinery, leading to the resistant phenotype of tumor cells in chemo/radiotherapy. HIF-1 may prevent apoptotic cell death by suppressing the intrinsic or extrinsic cell death pathway. HIF-1α is reported to inhibit pro-apoptotic proteins, such as Bax and caspase 3/8, and activate anti-apoptotic proteins, such as c-myc, survivin, STAT3, and transcription factor 4 (TCF4) [122]. The HIF-1-mediated inhibition of p53 activation in response to 5-fluorouracil (5-FU) is also reported, demonstrating the p53-dependent suppression of 5-FU-induced apoptosis by HIF-1 [123]. In addition, HIF-1α regulates the balance between NF-κB-associated pro- and anti-apoptosis, resulting in the resistance of pancreatic cancer cells to gemcitabine [124]. Alternatively, HIF-1 suppresses the extrinsic cell death pathway by blocking the binding of a death ligand to a death receptor on the cell surface, which permits the cells to tolerate higher amounts of chemotherapeutic harm before the activation of cell death pathways. For example, HIF-1 increased the expression of decoy receptors (DcR1 and DcR2), which then competed with DR4 and DR5 receptors for binding to the tumor necrosis factor-related apoptosis-inducing ligand (TRAIL), thereby neutralizing TRAIL toxicity [125]. Accordingly, HIF-1α is an important mediator of chemo/radioresistance in solid tumors by inhibiting apoptosis.

As described earlier (Section 3.1), autophagy is an essential pro-survival pathway in hypoxia conditions and has an emerging role in the development of resistance to anticancer therapy. For their survival against chemo/radiotherapy-induced apoptosis, tumor cells degrade cellular components through autophagic processes and reuse the products for metabolic biosynthesis and as energy sources. Numerous studies have demonstrated that the HIF-1α-mediated activation of autophagy causes chemo/radioresistance in tumor cells [126,127]. Autophagy-related proteins (ATGs), such as Beclin-1, and the microtubule-associated protein 1A/1B-light chain 3 (LC3) are critical proteins involved in autophagic regulation and the formation of autophagosome [128,129]. Cisplatin increased the protein levels of ATG5, Beclin-1, and LC3 in lung cancer cells, exhibiting features indicative of autophagy. Hypoxia was shown to robustly augment cisplatin-induced autophagy in a HIF-1-dependent manner, thereby leading to cisplatin resistance by decreasing its sensitivity [130]. Other studies also demonstrated that HIF-1α-induced autophagy could contribute to the cisplatin resistance of ovarian and lung cancer cells via the upregulation of Beclin-1 and LC3 [130,131]. Moreover, suppression of the HIF-1α/BNIP3/Beclin-1 signaling pathway inhibited autophagy and enhanced both gemcitabine-induced apoptosis and gemcitabine sensitivity in bladder cancer cells under hypoxic conditions [132]. Based on these observations, it has been proposed that targeting autophagy may be sufficient to restore lung cancer cell susceptibility to cisplatin [130]. Similarly, hypoxia-induced autophagy is reported to be involved in the radioresistance of human osteosarcoma and lung cancer cells by HIF-1α activation [133,134].

**Figure 3:**
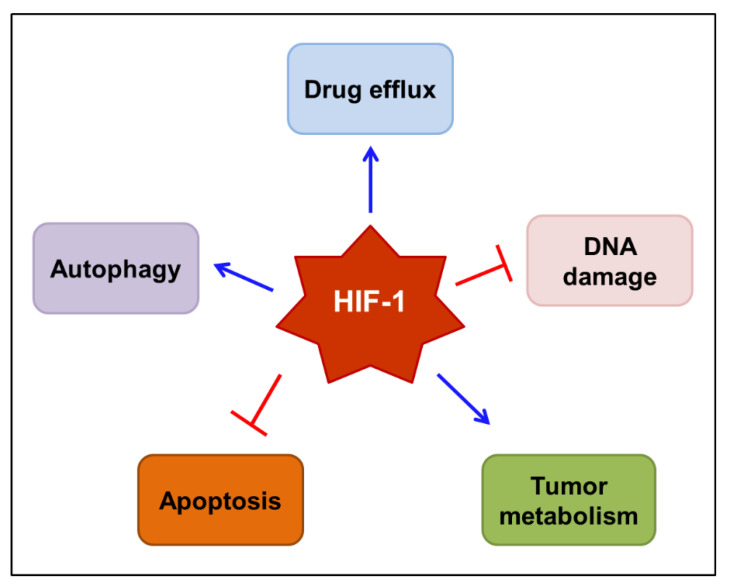
Proposed mechanisms by which HIF-1 mediates resistance to anticancer therapy. HIF-1 may mediate the resistance to chemo/radiotherapy by inducing drug efflux transporters, such as *MDR1*/P-gp, repairing DNA damage, reprogramming tumor metabolism, interrupting programmed cell death, including apoptosis, and augmenting autophagy. Blue arrows indicate stimulation, while blocked red arrows indicate inhibition. HIF-1—hypoxia-inducible factor-1; MDR1—multidrug resistance 1 protein; P-gp—P-glycoprotein.

Taken together, HIF-1 has an emerging role in the chemo/radioresistance of tumors. The complex mechanisms by which HIF-1 mediates resistance to anticancer therapies are gradually being elucidated. The mechanisms proposed to underlie HIF-1-mediated drug resistance are outlined in Figure 3. These include the induction of drug efflux transporters, such as *MDR1*/P-gp, the repairment of DNA damage, the reprogramming of tumor metabolism, the interruption of apoptosis, and the augmentation of autophagy. Therefore, it would be reasonable to use HIF-1 inhibitors to prevent the development of resistance to chemo/radiotherapy.

Numerous studies have demonstrated the possibility of preventing the development of resistance to chemo/radiotherapy using HIF inhibitors in combination with various anticancer drugs or radiation. For example, LW6, a well-designated HIF-1α inhibitor, was discovered as a potent inhibitor of BCRP, thereby preventing resistance to doxorubicin [135]. Similarly, LC478 inhibited the induction of P-gp, which resulted in an increased docetaxel absorption in a rat model of colorectal adenocarcinoma [136]. EZN-2208 (PEG-SN38) was found to overcome the ATP-binding cassette superfamily G member 2 (ABCG2)-mediated topotecan resistance by increasing the DNA damage repair in breast cancer 1 (BRCA1)-deficient mouse mammary tumors [137,138], while chetomin blocked the reprogramming of tumor metabolism in human malignant glioma cells to prevent hypoxic radioresistance [139]. Some HIF-1 inhibitors, such as PX-478, PMX290, and FK228, were shown to enhance the antitumor effects of chemo/radiotherapy by inducing apoptosis in pancreatic ductal adenocarcinoma (PDAC) and various other cancers [140,141,142,143,144,145,146,147,148,149,150,151]. Both drug efflux and apoptosis mechanisms are involved in the prevention of resistance to chemotherapy by other HIF-1 inhibitors, including YC-1, RAD001 (everolimus), SCH66336 (lonafarnib), and echinomycin [152,153,154,155,156,157]. Selected HIF-1 inhibitors demonstrated an ability to prevent chemo/radiotherapy resistance in various types of cancer or cancer cell lines and are summarized in Table 1.

### 4.2. Role of HIF-1 in Cancer-Related Pain

Cancer and anticancer therapies can often lead to physical and psychological burdens in patients. One of the serious burdens is cancer-related pain, affecting approximately 40% of patients with cancer [159]. Patients can experience pain in every stage of cancer until the end of life. It may interfere with their treatment process, lead to treatment refusal, and impact their survival [160]. Peripheral neuropathic cancer pain (PNCP) is one of the most complex conditions among cancer-related pain syndromes. PNCP syndromes can be either acute or chronic [161]. Acute PNCP is most frequently associated with cancer diagnostic or therapeutic interventions. Tissues, especially nerves, are directly injured by diagnostic approaches, resulting in pain. Chemo/radiotherapy induces acute PNCP at the beginning of treatment or as a side effect [161]. Chronic PNCP results from treatment complications or malignancy itself [161]. Currently, the molecular mechanisms by which chemo/radiotherapy induces PNCP to remain elusive. However, the pain level was shown to be closely related to the growth of the tumor and its microenvironment and the fact that hypoxic nerves are more vulnerable than normal nerves. In the long term, chronic hypoxia leads to fibrosis in perineural tissues and causes PNCP [162]. Therefore, the activation of HIF-1 may be involved in cancer-related pain, especially PNCP.

Bortezomib is a well-known anticancer drug that causes peripheral neuropathic pain as an unwanted side effect. Bortezomib has been demonstrated to induce aerobic glycolysis in sensory neurons, leading to the augmented extrusion of metabolites that sensitize primary afferents and causes pain [163,164]. Boyette-Davis et al. [163] found that the treatment of mice with bortezomib stabilized the expression of HIF-1α. Moreover, the bortezomib-induced neuropathic pain was abolished by the knockdown of HIF-1α expression, disruption of HIF-1α translation, or its binding to HREs. These results establish that the stabilization of HIF-1α expression is closely associated with the initiation of PNCP [165]. Strikingly, the phase I/II trial involving 72 patients with refractory MM, no patients developed severe peripheral neuropathy when using tanespimycin (17-allylamino-17-demethoxygeldanamycin: 17-AAG) in combination with bortezomib, indicating that HIF-1 inhibitors in combination with conventional anticancer therapy could reduce the therapy-induced pain and enhance the treatment efficacy [166].

A possible correlation between cancer-related pain and the expression levels of HIF-1 and VEGF was examined in patients with liver cancer. Before and after an intervention, such as treatment with analgesics, hepatic surgeries, or chemo/radiotherapy, the mRNA expression levels of HIF-1 and VEGF in the liver cancer group with pain were significantly higher than those in the group without pain. Furthermore, the HIF-1 and VEGF mRNA expression in the group with pain markedly increased after the intervention. A visual analog scale used to evaluate the pain level was positively correlated with the HIF-1 and VEGF expression in the group of liver cancer patients with pain before and after the intervention, suggesting that chemo/radiotherapy may result in pain via the activation of the HIF-1 pathway [167]. In an in vivo study using a metastatic bone cancer pain model, the downregulation of annexin A3, a highly expressed protein during bone cancer pain, alleviated pain by inhibiting the HIF-1/VEGF signaling pathway [168]. Altogether, increased HIF-1 and VEGF levels were closely correlated with therapeutic failure, possibly due to increased pain occurrence and pain levels. Thus, targeting the HIF-1 and VEGF pathways may provide a potential benefit for alleviating cancer-related pain.

Notably, the tumor-derived granulocyte-macrophage colony-stimulating factor (GM-CSF) has been reported to play an important role in the development of hypersensitive pain in tumor-affected areas [169]. The disruption of GM-CSF signaling by the downregulation of its receptor reduced the hypersensitive pain evoked by bone cancer in vivo [170]. GM-CSF and other hematopoietic factors are also shown to be expressed in the sensory nerves of peripheral tissues in pancreatic cancer [171]. The overexpression of GM-CSF induced nerve cell migration and was significantly correlated with pain in patients with PDAC. The expression levels of HIF-1α and GM-CSF in PANC-1 pancreatic cancer cells were increased when cultured in the hypoxic condition compared with those in the normoxic condition. Moreover, when HIF-1α was overexpressed in PANC-1 cells and MIA PaCa2 cells (human pancreatic carcinoma cells), GM-CSF mRNA and protein expression levels were also markedly increased, indicating that the hypoxia microenvironment could regulate the expression of GM-CSF through the overexpression of HIF-1α [172]. HIF-1α directly activates the transcription and expression of GM-CSF and mediates tumor−nerve interactions in PDAC. Therefore, agents targeting the HIF-1α/GM-CSF signaling might inhibit tumor−nerve interactions and help to alleviate the pain in PDAC patients [172].

Although HIF-1α is protective in terms of acute heat and cold pain in the early phase, the ongoing activation of injured neurons may promote the development of chronic neuropathic pain via the upregulation of HIF-1 target genes, including *GCH1* encoding GTP cyclohydrolase 1 (GTPCH) and its product tetrahydrobiopterin (BH4), which further drives NO production. Therefore, the therapeutic interruption of HIF-1/GTPCH/BH4 activation may reduce neuropathic pain. Due to the dual roles of HIF-1 in pain regulation, the drugs targeting HIF-1 may cause side effects in terms of heat and cold pain sensitivity. However, in patients with neuropathy associated with cancer treatment, HIF-1 inhibitors may provide a combination of tumor growth inhibition and pain reduction [173].

**Figure 4:**
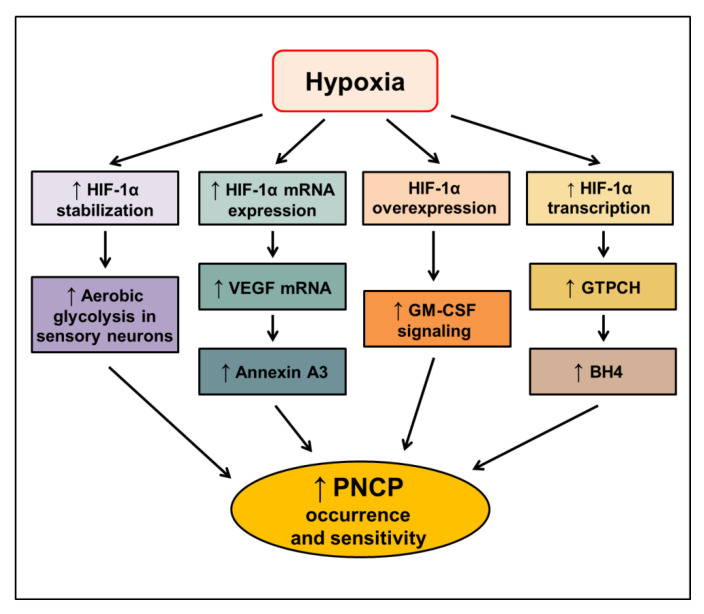
Proposed signaling pathways inducing peripheral neuropathic cancer pain (PNCP) through HIF-1α in hypoxic conditions. HIF—hypoxia-inducible factor-1, VEGF—vascular endothelial growth factor; GM-CSF—granulocyte-macrophage colony-stimulating factor; GTPCH—GTP cyclohydrolase 1; BH4—tetrahydrobiopterin. The upward arrow indicates an increase in the respective signal.

Altogether, the activation of HIF-1 is closely associated with cancer-related pain, especially PNCP. The proposed signaling pathways mediating the increased occurrence and sensitivity of PNCP through HIF-1α in hypoxic conditions are elucidated (Figure 4). However, the molecular mechanisms by which HIF-1 regulates cancer-related pain remain poorly understood. Further studies should address the role of HIF-1 in cancer pain to provide advantages for targeting HIF-1 as a novel anticancer approach.

## 5. Current Clinical Status of HIF-1 Inhibitors as Potential Anticancer Therapy

**Figure 5:**
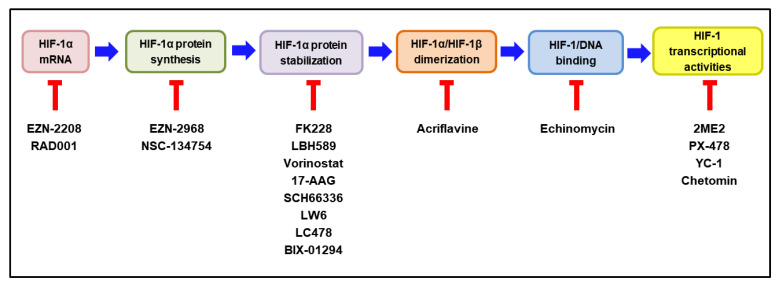
Molecular mechanisms of HIF-1 inhibitors. The steps required to activate HIF-1 are illustrated in colored boxes, and the representative HIF-1 inhibitors acting at each step are listed under the respective box. HIF—hypoxia-inducible factor-1. The blocked red arrows indicate inhibition.

According to the extensive involvement of HIF-1 in tumor progression, many HIF-1 inhibitors have been developed and evaluated as potential anticancer drugs in pre-clinical and clinical studies [14,174,175]. HIF-1 inhibitors can affect the expression or function of HIF-1 through a wide range of molecular mechanisms, including the inhibition of HIF-1α mRNA, HIF-1α protein synthesis, HIF-1α stabilization, HIF-1α/HIF-1β dimerization, HIF-1/DNA binding, and HIF-1 transcriptional activities [176,177]. Intriguingly, MO-460, an analog of a benzofuran-based natural product (R)-(-)-moracin-O, has been reported to inhibit HIF-1α accumulation by the previously unidentified mechanism. It was demonstrated to interact with the heterogeneous nuclear ribonucleoprotein A2/B1 (hnRNPA2B1), thereby inhibiting the initiation of HIF-1α translation [178]. The molecular mechanisms of the representative HIF-1 inhibitors are delineated in Figure 5. Some of these HIF-1 inhibitors have been evaluated for their efficacy and safety in clinical trials. The current clinical status of these inhibitors, either as a monotherapy or in combination with other anticancer therapies, is discussed below.

### 5.1. HIF-1 Inhibitor Decreasing HIF-1α mRNA Expression

#### EZN-2208

EZN-2208, a polyethylene glycol (PEG)-coated SN-38 (PEG-SN38), which is an active metabolite of irinotecan, inhibits the HIF-1 pathway through the suppression of HIF-1α at the mRNA level [151]. By this mechanism, it was shown that to reduce the expression of MMP2, VEGF1, GLUT1, GLUT3, and TGFβ1, is to subsequently inhibit the angiogenesis of tumor cells [151]. The clinical tolerability and antitumor activity of EZN-2208, either as a monotherapy or in combination with bevacizumab, has been demonstrated in a phase I study in patients with refractory solid tumors [179,180]. In a randomized phase II study, patients with metastatic CRC who previously received 5-FU, oxaliplatin, and irinotecan were assigned with EZN-2208 monotherapy for patients with Kirsten rat sarcoma viral oncogene homolog (*KRAS*)-mutant tumors. Overall, the response rate and progression-free survivals were 0% and 1.8 months, respectively. The overall response rate and progression-free survivals were 10.7% and 4.9 months, respectively, in patients with *KRAS*-wild type tumors who received EZN-2208 plus cetuximab, whereas the corresponding values were 14.3% and 3.7 months in the patients who received irinotecan plus cetuximab [181]. These results demonstrate that EZN-2208, either as a monotherapy or in combination with cetuximab, was well-tolerated in patients with refractory CRC and that the overall survival and progression-free survival of patients with *KRAS*-wild type tumors were similar when treated with cetuximab plus irinotecan or EZN-2208.

### 5.2. HIF-1 Inhibitor Decreasing HIF-1α Protein Synthesis

#### EZN-2968

EZN-2968 is a locked nucleic acid oligonucleotide that inhibits HIF-1α protein synthesis by blocking the translation of HIF-1α mRNA. The inhibition of HIF-1α by EZN-2968 reduced tumor growth by inhibiting cell proliferation through a delayed progression of the S-phase, which might be caused by the shift to a mitochondrial oxidative metabolism [182]. In a phase I study involving 10 patients with refractory solid tumors, one patient with a duodenal neuroendocrine tumor showed prolonged stabilization of the disease for 24 weeks. Reduced levels of the HIF-1α protein and the mRNA of some target genes were observed in two patients [183]. Although the trial has been closed prematurely, it provides preliminary proof that the modulation of HIF-1α synthesis and its target genes may exhibit potential anticancer activity.

### 5.3. HIF-1 Inhibitors Decreasing HIF-1α Stabilization

Drugs that decrease HIF-1α stabilization by inducing its degradation include histone deacetylase inhibitors, such as FK228, LBH589, and vorinostat; heat shock protein 90 inhibitors include 17-AAG and farnesyl transferase inhibitors, such as SCH66336 (Figure 5).

#### 5.3.1. FK228 (Romidepsin)

Romidepsin was shown to inhibit hypoxia-responsive angiogenesis factors, such as VEGF and the basic fibroblast growth factor, through the suppression of HIF-1α stabilization [184,185]. Romidepsin (8 mg/m^2^) plus erlotinib was well tolerated, showed disease control, and exhibited beneficial effects in a phase I study involving 17 patients with advanced NSCLC [186]. However, a phase II trial of single romidepsin treatment showed limited activity in the squamous cell carcinoma of the head and neck (SCCHN) [187].

#### 5.3.2. LBH589 (Panobinostat)

Panobinostat caused apoptosis mediated by chromatin fragmentation, the activation of caspases-3 and 7, and PARP via HIF-1α destabilization in cisplatin-resistant lung cancer cells [188]. In a phase I study, the combination of carfilzomib with panobinostat proved a safe and effective treatment option for patients with relapsed/refractory MM [189,190]. To compare the clinical tolerability of panobinostat in combination with other anticancer drugs, 768 patients with refractory MM were enrolled in a randomized, double-blind phase III trial. Patients received 21-day cycles of a placebo or panobinostat (20 mg, oral), both in combination with bortezomib (1.3 mg/m^2^, intraperitoneal) and dexamethasone (20 mg, oral). The median follow-up was 6.47 months in the panobinostat group and 5.59 months in the placebo group. The median progression-free survival was significantly longer in the panobinostat group than in the placebo group (11.99 months vs. 8.08 months) [191]. These results suggest that panobinostat could be a useful addition to conventional anticancer therapies for patients with refractory MM.

#### 5.3.3. Vorinostat

Vorinostat was the first histone deacetylase inhibitor approved by the United States Food and Drug Administration (FDA) for the treatment of cutaneous T-cell lymphoma. Vorinostat potently inhibited HIF-1α stabilization via the acetylation of its associated chaperone heat shock protein 90 (Hsp90) and subsequently suppressed the downstream molecules, including EPO, GLUT 1, and VEGF [192]. The combination of vorinostat with temozolomide and radiation therapy had acceptable tolerability in newly diagnosed glioblastoma in phase I and phase II trials involving 15 and 107 patients, respectively [193]. In another phase II study involving 32 patients with advanced melanoma, 16 had stable disease, while 14 had progressive disease as their best response. Two patients with cutaneous melanoma that scored stable disease had early unconfirmed partial responses with subsequent progression. Patients with stable disease or partial response (*n* = 18) had a median progression-free survival of 5 months [194]. However, a double-blind, randomized, placebo-controlled phase III trial reported that vorinostat did not improve overall survival and was not recommended for patients with advanced malignant pleural mesothelioma [195].

#### 5.3.4. 17-AAG (Tanespimycin)

Tanespimycin was found to inhibit Hsp90-implicated HIF-1α signaling, including HIF-1α destabilization and VEGF secretion, consequently suppressing angiogenesis and tumor growth [196]. In a phase II study assessing the efficacy of 17-AAG and its toxicity profile in patients with metastatic, hormone-refractory prostate cancer, 17-AAG did not show any anticancer activity regarding the prostate-specific antigen (PSA) response. Due to an insufficient PSA response and severe toxicity, enrollment was stopped at the end of the first stage of the study design [197].

However, tanespimycin in combination with bortezomib was well tolerated in thephase I/II study. Among 67 efficacy-evaluable patients with refractory MM, there were two complete responses (3%) and eight partial responses (12%), with an overall objective response rate of 27%, including eight minimal responses (12%) [166]. Although the evaluation of 17-AAG as a single agent is not warranted, 17-AAG might have promising efficacy in combination with other anticancer drugs.

#### 5.3.5. SCH66336 (Lonafarnib)

It has been revealed that the antiangiogenic activities of SCH66336 are mediated by the disrupted connection of HIF-1α and Hsp90 and destabilization of HIF-1α, leading to a decreased expression of HIF-1α [198]. SCH66336 has shown marked antitumor activities as a monotherapy in a phase Ib study of SCCHN (https://clinicaltrials.gov/ct2/show/NCT00038584 accessed on 30 October 2018). Then, a phase II study was conducted to examine its efficacy and safety in patients with recurrent, refractory SCCHN. However, due to no objective responses observed in the first 15 patients, further evaluation of SCH66336 in refractory SCCHN was not planned [199].

In another phase II trial of SCH66336, patients with metastatic CRC refractory to 5-FU and irinotecan received SCH66336 as a twice-daily oral administration. Although three patients showed stable disease for several months, no objective responses were observed. The administration of SCH66336 was accompanied by gastrointestinal toxicity. The future development of this drug is not recommended as a monotherapy for this disease [200].

However, the combination treatment of SCH66336 with paclitaxel was well-tolerated, with minimal toxicity in patients with taxane-refractory/resistant metastatic NSCLC. The evaluation of this combination therapy in additional clinical trials is warranted [155].

### 5.4. HIF-1 Inhibitor Decreasing HIF-1α/HIF-1β Dimerization

#### Acriflavine

Acriflavine binds to the PAS-B domain of HIF-1α, blocking its interaction with HIF-1β, which leads to the suppression of tumor growth and tumor vascularization [201]. Moreover, acriflavine also enhances the antitumor activity of sunitinib in a breast cancer model [202]. However, there is no study investigating its efficacy in clinical conditions.

### 5.5. HIF-1 Inhibitor Decreasing HIF-1α/DNA Binding

#### Echinomycin

Echinomycin, a small-molecule inhibitor of HIF-1/DNA binding, did not show significant antitumor activity in phase II studies in patients with ovarian cancer, breast cancer, renal cell carcinoma, or CRC [203,204,205,206].

### 5.6. HIF-1 Inhibitor Decreasing HIF-1α Transcriptional Activity

#### 2-Methoxyestradiol (2ME2, Panzem)

2ME2, a natural metabolite of estradiol inhibiting transcriptional activities of HIF-1α, showed strong antiangiogenic and antiapoptotic effects on cancer cells [207]. Sixty MM patients enrolled in the phase II clinical study were treated with 2ME2, but no partial responses have been observed so far. The lack of objective responses to the 2ME2 treatment may be due to the inadequate dosage of the drug, as indicated by the low plasma level [208]. Another phase II trial of 2ME2 in 50 patients with metastatic prostate cancer was also terminated after the enrollment of 21 patients due to the futility and lack of objective response to the treatment [209].

There were no objective responses in a phase II trial of 2ME2 in patients with advanced, platinum-resistant ovarian cancer, but 2ME2 was still well-tolerated, and seven patients had stable disease as the best response. Of those, two patients had stable disease for greater than 12 months. The rate of clinical benefit was 31.3%. Fairly stable plasma levels of 2ME2, ranging within the predicted therapeutic window, were observed [210].

In another phase II clinical trial, 2ME2 was used in combination with bevacizumab for the treatment of 31 patients with a locally metastatic carcinoid tumor. Among them, 21 patients showed a reduction in tumor size, and the median progression-free survival was 11.3 months, supporting the concept that this regimen had some degree of antitumor activity when used in combination with other anticancer agents [211].

In summary, the antitumor activities of HIF-1 inhibitors have been well-demonstrated in many preclinical studies [177]. Despite the limited efficacy of various HIF-1 inhibitors in clinical trials, vorinostat has been reported to exert clinically recognizable benefits in the treatment of melanoma [194]. However, many HIF-1 inhibitors have failed to show efficacy in clinical trials. To improve the therapeutic efficacy of HIF inhibitors and reduce drug resistance and cancer-related pain, the development of combination therapies may be necessary. In fact, several HIF-1 inhibitors administered in combination with the other conventional therapeutic agents for the treatment of refractory cancers suggested better outcomes. Further preclinical and clinical studies are warranted to elucidate the promising HIF-1 inhibitors for combination therapies. The current status of clinical trials evaluating HIF-1α inhibitors as potential anticancer therapies is summarized in Table 2.

## 6. Conclusions and Future Perspectives

The roles of hypoxia in biological hallmarks of cancer involve complicated signaling centered on its main mediator, HIF-1. Evidence has shown that HIF-1 significantly impacts cancer progression, from tumor cell proliferation to angiogenesis and tumor metastasis. Clinical trials indicate that some HIF-1 inhibitors have potential either as a monotherapy or in combination with other conventional anticancer therapies. Further studies prove that hypoxia drives not only tumor biology but also the tumor microenvironment that contributes to the development of resistance to cancer treatment. We also provided a comprehensive view of the underlying molecular mechanisms by which HIF-1 regulates chemo/radioresistance. Furthermore, a novel insight into the correlation between cancer-related pain and HIF-1 activation was addressed in this review. Several HIF-1 inhibitors are found to enhance the therapeutic efficacy of anticancer drugs, overcome resistance to anticancer drugs, and prevent cancer-related pain. Each therapy has advantages and limitations, and each cancer type has different characteristics. Thus, the identification of hypoxic markers and genomic analyses are urgently required to allow HIF-1 inhibitors to be tailored to specific cancer types and individual patients. A more detailed understanding of HIF-1 regulation in tumor progression would improve cancer care.

## Figures and Tables

**Table 1:** Selected HIF-1 inhibitors were demonstrated to prevent chemo/radiotherapy resistance in various types of cancer or cancer cell lines.

HIF-1 Inhibitors	Anticancer Therapy	Cancer Type/ Cell Line	Mechanism for Prevention of Resistance	References
LW6	Mitoxantrone Doxorubicin	Breast cancer	Drug efflux	[135]
LC478	Docetaxel	Colorectal adenocarcinoma	Drug efflux	[136]
EZN-2208 (PEG-SN38)	Topotecan	Breast cancer	DNA damage repair	[137,138]
Chetomin	Radiation	Glioma	Metabolism	[139]
PX-478	Radiation Gemcitabine	Pancreatic cancer	Apoptosis	[140,141]
PMX290	5-Fluorouracil	Colon adenocarcinoma	Apoptosis	[142]
FK228 (Romidepsin)	Temozolomide	Glioma	Apoptosis	[143]
BIX-01294	TRAIL	Hepatocellular carcinoma	Apoptosis	[144]
LBH589	Cisplatin Bortezomib Osimertinib	Ovarian cancer Multiple myeloma Lung cancer	Apoptosis	[145] [146] [147]
Vorinostat	Paclitaxel Doxorubicin Bortezomib	Breast cancer Neuroblastoma Mesothelioma	Apoptosis	[148] [149] [150]
NSC-134754	Cisplatin Doxorubicin	Osteosarcoma	Apoptosis	[151]
YC-1	Gefitinib Cisplatin	Lung cancer Oral cancer	Drug efflux Apoptosis	[152] [153]
RAD001 (Everolimus)	Cisplatin	Gastric cancer	Drug efflux Apoptosis	[154]
SCH66336 (Lonafarnib)	Paclitaxel Cisplatin	Lung cancer Melanoma	Drug efflux Apoptosis	[155] [156,157]
Echinomycin	Hormone	Prostate cancer	n.d.	[158]

TRAIL—tumor necrosis factor-related apoptosis-inducing ligand; n.d.—not determined.

**Table 2:** Current clinical status evaluating HIF-1α inhibitors as potential anticancer therapies.

Mechanism of HIF-1 Inhibition	HIF-1 Inhibitor	Phase	Cancer Type	Reference
Decreasing HIF-1α mRNA expression	EZN-2208 (PEG-SN38)	Phase I Phase II	Refractory solid tumors Metastatic colorectal cancer	[179,180] [181]
Decreasing HIF-1α protein synthesis	EZN-2968	Phase I	Refractory solid tumors	[183]
Decreasing HIF-1α stabilization	FK228 (Romidepsin)	Phase I Phase II	NSCLC Head and neck cancer	[186] [187]
	LBH589 (Panobinostat)	Phase I Phase I Phase III	MM Refractory solid tumors Refractory MM	[189] [190] [191]
	Vorinostat	Phase I/II Phase II Phase III	Glioblastoma Advanced melanoma Mesothelioma	[193] [194] [195]
	17-AAG (Tanespimycin)	Phase II Phase I/II	Prostate cancer Refractory MM	[197] [166]
	SCH66336 (Lonafarnib)	Phase II Phase II Phase II	SCCHN Lung carcinoma Colorectal cancer	[199] [200] [155]
Decreasing HIF-1/ DNA binding	Echinomycin	Phase II Phase II Phase II Phase II	Ovarian cancer Breast cancer Renal cell carcinoma Colorectal cancer	[203] [204] [205] [206]
Decreasing HIF-1α protein synthesis and transcriptional activity	2ME2	Phase II Phase II Phase II Phase II	MM Prostate cancer Ovarian cancer Carcinoid tumors	[208] [209] [210] [211]

HIF-1—hypoxia-inducible factor-1; 17-AAG—17-allylamino-17-demethoxygeldanamycin; 2ME2—2-methoxyestradiol; NSCLC—non-small cell lung carcinoma; SCCHN—squamous cell carcinoma of the head and neck; MM—multiple myeloma. (For details, please see the text.).
